# Pentavalent lanthanide nitride-oxides: NPrO and NPrO^–^ complexes with N≡Pr triple bonds†
†Electronic supplementary information (ESI) available. See DOI: 10.1039/c7sc00710h


**DOI:** 10.1039/c7sc00710h

**Published:** 2017-03-15

**Authors:** Shu-Xian Hu, Jiwen Jian, Jing Su, Xuan Wu, Jun Li, Mingfei Zhou

**Affiliations:** a Beijing Computational Science Research Center , Beijing 100094 , China; b Department of Chemistry and Key Laboratory of Organic Optoelectronics & Molecular Engineering of Ministry of Education , Tsinghua University , Beijing 100084 , China . Email: junli@tsinghua.edu.cn; c Collaborative Innovation Center of Chemistry for Energy Materials , Department of Chemistry , Shanghai Key Laboratory of Molecular Catalysis and Innovative Materials , Fudan University , Shanghai 200433 , China . Email: mfzhou@fudan.edu.cn

## Abstract

The neutral molecule NPrO and its anion NPrO^–^ are produced *via* co-condensation of laser-ablated praseodymium atoms with nitric oxide in a solid neon matrix. Combined infrared spectroscopy and state-of-the-art quantum chemical calculations confirm that both species are pentavalent praseodymium nitride-oxides with linear structures that contain Pr≡N triple bonds and Pr=O double bonds. Electronic structure studies show that the neutral NPrO molecule features a 4f^0^ electron configuration and a Pr(v) oxidation state similar to that of the isoelectronic PrO_2_^+^ ion, while its NPrO^–^ anion possesses a 4f^1^ electron configuration and a Pr(iv) oxidation state. The neutral NPrO molecule is thus a rare lanthanide nitride-oxide species with a Pr(v) oxidation state, which follows the recent identification of the first Pr(v) oxidation state in the PrO_2_^+^ and PrO_4_ complexes (*Angew. Chem. Int. Ed.*, 2016, **55**, 6896). This finding indicates that lanthanide compounds with oxidation states of higher than +IV are richer in chemistry than previously recognized.

## Abstract 2

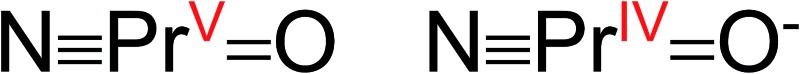
The neutral molecule NPrO and its anion NPrO^–^ are characterized to be linear pentavalent praseodymium nitride-oxides that possess Pr≡N triple bonds and Pr=O double bonds.

## Introduction

Due to the vital role of lanthanide elements in modern technology, which includes their applications in rare-earth catalysis, electronics, wind power and magnets, *etc.*, lanthanide chemistry has attracted intensive attention over the past few decades.^1^ Although high oxidation states of +VIII and even +IX have been reported for some main group and transition metal elements in the periodic table,^2^ the oxidation states of f-elements are much more complicated.^3^ The chemistry of lanthanides is dominated by the low-valent +III or +II oxidation states due to the chemical inertness of the 4f valence electrons.^4^ Until recently the highest known oxidation state of the whole lanthanide series had been +IV, as is found commonly in Ce as well as in Pr, Nd, Tb and Dy. Among all of the elements in the lanthanide series, it has been postulated since the early 1900s that praseodymium, with five valence electrons and the lowest fifth ionization energy, could be oxidizable beyond the +IV oxidation state.^5^ It had been reported that Y_2_O_3_ could promote the oxidation of praseodymium to the +V oxidation state by forming the compound YPrO_4_.^6^ However, the claimed Pr(v) state in oxide solids was firmly refuted in 1950.^7^ About 50 years later, the Pr(v) oxidation state was again suggested to exist in the gas-phase PrO_3_^–^ anion,^8^ but a later theoretical study indicated that PrO_3_^–^ is in fact a highly multi-configurational Pr(iv) species with a biradical feature.^9^ Besides the oxides, praseodymium pentafluoride, PrF_5_, was predicted to be unstable toward dissociation into PrF_4_ and PrF_3_ in the gas phase, and was not formed in the reaction of laser-ablated praseodymium atoms with F_2_ in solid noble gas matrices.4*d* No pentavalent lanthanide compounds with +V oxidation states had been experimentally confirmed until recently when the praseodymium oxide species PrO_4_ and PrO_2_^+^ were prepared in the gas phase and in solid noble-gas matrices.^10^ Combined infrared spectroscopy and advanced quantum chemistry studies revealed that these praseodymium oxide species feature the unprecedented Pr(v) oxidation state, thus demonstrating that the +V oxidation state is viable for lanthanide elements in a suitable coordination environment.^10^ So far, no other lanthanide compounds have been found to possess the Ln(v) oxidation state.

Herein, we report a combined experimental and theoretical study on the neutral NPrO molecule and its anion NPrO^–^ in solid neon. Combined matrix-isolation infrared absorption spectroscopy and sophisticated quantum chemistry studies reveal that both species are linear pentavalent compounds with Pr≡N triple bonds and Pr=O double bonds, and that the neutral NPrO molecule, which is isoelectronic to the PrO_2_^+^ cation, is also a pentavalent praseodymium species with a Pr center in the highest +V oxidation state. The NPrO species thus provides additional evidence that lanthanides can form complexes with oxidation states higher than IV.

## Experimental details and computational methods

Praseodymium nitride-oxide species were prepared by reactions of praseodymium atoms and nitric oxide in solid neon, and were studied by infrared absorption spectroscopy as described in detail previously.^11^ A fundamental 1064 nm Nd : YAG laser (Continuum, Minilite II; 10 Hz repetition rate) was used to evaporate praseodymium atoms from a rotating praseodymium metal target, which were co-deposited with NO reagent gas in excess neon onto a cryogenic window maintained at 4 K by means of a closed-cycle helium refrigerator. NO/Ne mixtures were prepared in a stainless steel vacuum line using a standard manometric technique. NO (Dalian DT, >99.9%) and isotopic-labeled ^15^NO (Cambridge isotope laboratories Inc, 98%) were used without further purification. The as-deposited samples were subjected to annealing and photolysis experiments to initiate diffuse and photo-induced reactions. The infrared absorption spectra of the products were recorded in the mid-infrared region (4000–450 cm^–1^) using a Bruker Vertex 80V spectrometer at a resolution of 0.5 cm^–1^ using a liquid nitrogen cooled HgCdTe (MCT) detector.

Quantum chemical calculations were performed at the density functional theory (DFT) and wavefunction theory (WFT) levels to gain insight into the geometries, electronic structures, bonding and oxidation states of the observed species. The hybrid B3LYP functional^12^ implemented in the Gaussian 09 program^13^ and the *ab initio* single-reference WFT method of coupled-clusters with singles, doubles and perturbative triples (CCSD(T))^14^ implemented in the MOLPRO^15^ program were used to optimize the geometry structures and to calculate the harmonic vibrational frequencies. Considering the relatively strong relativistic effects on Pr, the ECP28MWB pseudopotential and ECP28MWB_ANO basis set for the Pr atom and the Dunning’s correlation consistent basis set with polarized triple-zeta plus diffuse functions (aug-cc-pVTZ) for the N and O atoms were employed.^16^

In addition, multi-configurational complete active space self-consistent-field theory and complete active space with second-order perturbation theory (CASSCF/CASPT2) methods^17^ implemented in the MOLCAS 8.0 software package^18^ were also employed to check whether the systems had unexpected multi-reference character and to obtain the correct ground electronic states for the observed compounds. In the CASSCF/CASPT2 calculations, scalar-relativistic effects were taken into account through the use of the second-order Douglas–Kroll–Hess (DKH2) approximation.^19^ Relativistic all-electron ANO-RCC-VDZP basis sets^20^ were used for all elements in the CASSCF and CASPT2 calculations.

Electronic structure and bonding analyses were performed at the B3LYP level using the ADF 2013 program.^21^ Herein, relativistic effects were taken into account through the zero-order regular approximation (ZORA).^22^ Slater basis sets with the quality of triple-ζ plus two polarization functions (TZ2P)^23^ were used with the frozen core approximation and applied to the inner shells of [1s^2^–4d^10^] for Pr, and [1s^2^] for O and N. Kohn–Sham molecular orbital (MO) analysis and calculations of Mayer bond order indexes^24^ were also carried out using this program. Natural bond orbital analysis was carried out using NBO 6.0 in the Gaussian 09 program.^25^

## Results and discussion

**Fig. 1:**
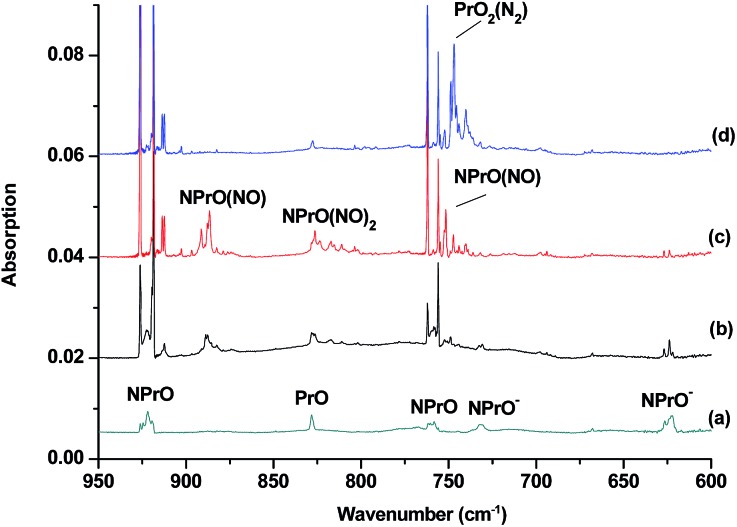
Infrared spectra in the 950–600 cm^–1^ region upon co-deposition of praseodymium atoms with 0.025% NO in neon. (a) After 30 min of sample deposition at 4 K, (b) after annealing at 10 K, (c) after annealing at 12 K, and (d) after 15 min of *λ* > 800 nm light irradiation.

**Fig. 2:**
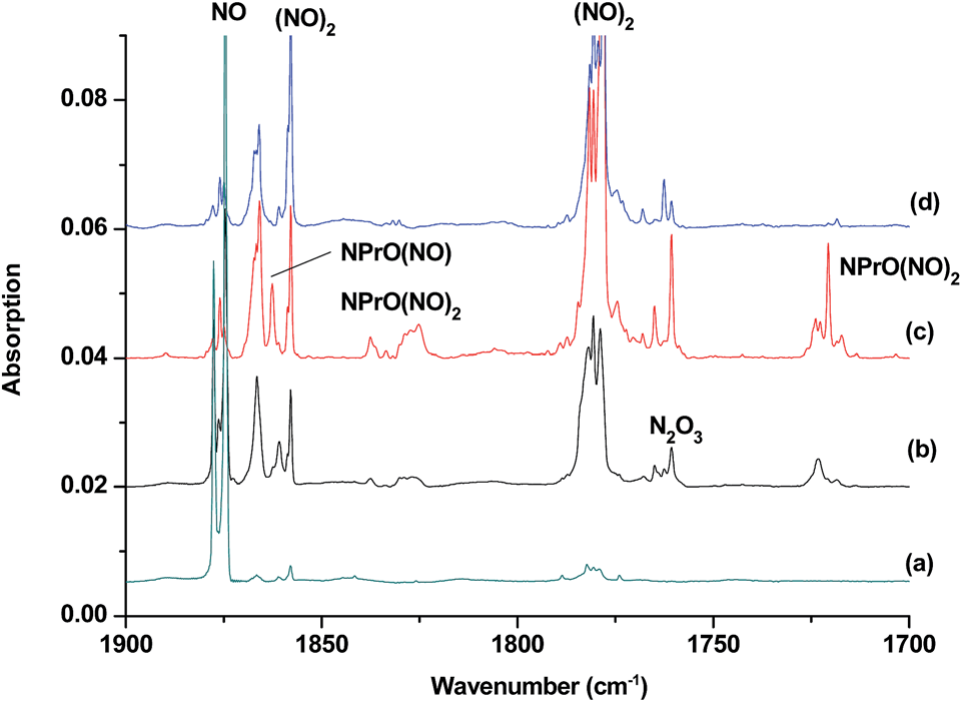
Infrared spectra in the 1900–1700 cm^–1^ region upon co-deposition of praseodymium atoms with 0.025% NO in neon. (a) After 30 min of sample deposition at 4 K, (b) after annealing at 10 K, (c) after annealing at 12 K, and (d) after 15 min of *λ* > 800 nm light irradiation.

**Fig. 3:**
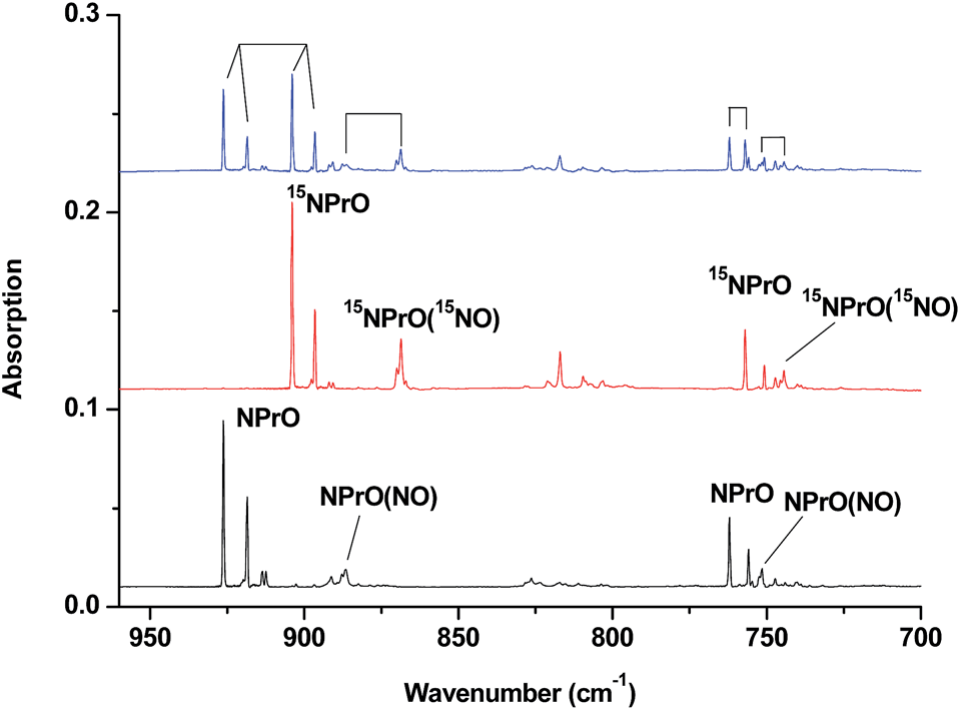
Infrared spectra in the 960–700 cm^–1^ region upon co-deposition of praseodymium atoms with isotopic-labeled NO in excess neon. The spectrum was recorded after annealing at 12 K. (a) 0.025% ^14^NO, (b) 0.025% ^15^NO, and (c) 0.025% ^14^NO + 0.025% ^15^NO.

The reaction products from the co-deposition of laser-ablated praseodymium atoms with nitric oxide in excess argon were previously studied using Fourier transform infrared absorption spectroscopy, which indicated the formation of NPrO and NPrO^–^ species in a solid argon matrix but did not address their oxidation states.26*a* A recent investigation indicated that the praseodymium dioxide cation is able to coordinate five noble gas atoms and form noble gas complexes in solid noble gas matrices,^10^ which is similar to what is observed for early transition metal and actinide elements.^27^ In order to minimize the matrix effect, the much more inert element neon is used as the matrix in the present study. The infrared spectra in selected frequency regions of the co-deposition of laser-ablated praseodymium atoms with 0.025% NO in neon are shown in Fig. 1 and 2. Besides the NO, (NO)_2_, (NO)_2_^+^ and (NO)_2_^–^ absorptions,^28^ which are common for laser-ablated metal atoms reacting with nitric oxide, the as-deposited sample exhibits a praseodymium-dependent absorption at 828.3 cm^–1^. This band can be attributed to diatomic PrO absorption, which is observed at 817.0 cm^–1^ in solid argon and at 826 cm^–1^ in the gas phase.^8^,^29^ Weak absorptions at 730.9 and 623.9 cm^–1^ were also observed upon sample deposition, sharpened and decreased upon sample annealing at 10 and 12 K, and diminished under *λ* > 800 nm light irradiation. Three additional groups of absorptions were produced when the sample was annealed at high temperatures (10 and 12 K) at the expense of NO absorption. The first group involves two doublet absorptions at 926.2/918.5 and 762.2/755.9 cm^–1^, which slightly decreased under *λ* > 800 nm irradiation. The second and third groups each involve three absorptions at 1862.6, 886.6 and 751.6 cm^–1^ and 1825.2, 1720.6 and 826.3 cm^–1^, respectively. The latter two groups of absorptions were destroyed upon *λ* > 800 nm light irradiation, during which absorption at 747.3 cm^–1^ was produced, which can be assigned to the antisymmetric OPrO stretching vibration of the weakly perturbed PrO_2_ complex, as this absorption shows no shift with ^15^NO with a band position very close to that of PrO_2_.^8^,^29^ The experiments were repeated under the same conditions using the ^15^NO and ^14^NO + ^15^NO samples. Selected regions of the isotopic spectra are shown in Fig. 3 and the product absorptions are listed in Table 1 and S1 of the ESI.† For comparison, argon matrix experiments were also performed. The spectra are shown in Fig. S1–S3,† and are quite similar to those previously reported.26*a*

**Table 1:** Comparison between the observed and calculated vibrational frequencies (cm^–1^) of NPrO and NPrO^–^

		NPrO		NPrO^–^	
		Pr–N stretch	Pr–O stretch	Pr–N stretch	Pr–O stretch
Experimental	Ne	926.2/918.5^*a*^	762.2/755.9^*a*^	730.9	623.9
	Ar	901.0	742.2	718.2	612.3
B3LYP ^*b*^		1021.1(378)	822.1(194)	813.9(593)	636.9(440)
CCSD(T)		960.1	788.5	767.8	613.7
CASPT2 ^*b*^		896.8(401)	735.6(97)	813.9(213)	645.7(303)

^*a*^Two site absorptions.

^*b*^The IR intensities are listed in parentheses in km mol^–1^.

The 926.2/918.5 and 762.2/755.9 cm^–1^ absorptions are assigned to the NPrO molecule trapped in solid neon in two trapping sites. The upper doublet shifted to 903.9/896.6 cm^–1^ with ^15^NO. The observed quite large ^15^N-isotopic shift indicates that this mode is mainly a Pr–N stretching vibration. The low doublet shifted to 757.0/750.7 cm^–1^ with ^15^NO, and the quite small ^15^N-isotopic shift implies that this mode is largely due to a Pr–O stretching vibration. Analysis of the spectrum obtained from the experiment using the mixed ^14^NO + ^15^NO sample confirms the involvement of only one N atom and one O atom in this molecule. The two stretching vibrational modes of NPrO were observed at 900.8 and 742.0 cm^–1^ in solid argon,26*a* and these were red-shifted from those in solid neon by 25.4 and 20.2 cm^–1^, respectively, thus indicating significant argon matrix effects that are non-negligible. The Pr–N stretching frequency of NPrO is higher than that of diatomic PrN, which was reported at 857.9 cm^–1^ in solid argon.26*b* In contrast, the Pr–O stretching frequency of NPrO is lower than that of diatomic PrO (828.3 cm^–1^ in Ne and 817.0 cm^–1^ in Ar). The NPrO absorptions increased markedly upon annealing in both neon and argon matrices, indicating that the ground state praseodymium atoms can insert into the N–O bond of nitric oxide spontaneously with negligible activation energy required.

The rather weak 730.9 and 623.9 cm^–1^ absorptions observed right after sample deposition correspond to the absorptions observed at 718.2 and 612.3 cm^–1^ in solid argon, which were attributed to the NPrO^–^ anion species.26*a* The 623.9 cm^–1^ absorption shows almost no shift with ^15^NO, thus indicating that this band is a pure Pr–O stretching mode. In contrast, the 730.9 cm^–1^ band shifted to 709.2 cm^–1^ with ^15^NO, and the isotopic ^14^N/^15^N frequency ratio of 1.0306 implies that it is a Pr–N stretching mode. The argon-to-neon shifts of 12.7 and 11.6 cm^–1^ are only about half of those of the neutral NPrO, thus suggesting that the NPrO^–^ anion is less affected by the noble gas atoms. The NPrO^–^ anion is presumably formed *via* electron capture of the neutral NPrO molecule during the co-condensation process. It is well-known that laser ablation of a metal target can produce not only neutral metal atoms but also metal cations and electrons.^30^ The adiabatic electron affinity of NPrO is calculated to be 7.1 kcal mol^–1^ at the CCSD(T) level of theory (overestimated as 26.9 kcal mol^–1^ with B3LYP), which is consistent with the experimental observation that the NPrO^–^ anion is photobleached upon *λ* > 800 nm light irradiation.

The 1862.6, 886.6 and 751.6 cm^–1^ absorptions are assigned to an NPrO(NO) complex. The upper mode shifted to 1829.8 cm^–1^ with ^15^NO, with the band position and isotopic shift indicating a terminally bound nitrosyl stretching vibration. The 886.6 and 751.6 cm^–1^ bands are assigned to the Pr–N and Pr–O stretching modes, respectively, which are slightly red-shifted from those of the NPrO molecule. The 1825.2, 1720.6 and 826.3 cm^–1^ absorptions are assigned to an NPrO(NO)_2_ complex, with the two upper absorptions corresponding to N–O stretching vibrations. The spectrum of the mixed ^14^NO + ^15^NO sample indicates that two equivalent NO subunits are involved in these two modes. The 826.3 cm^–1^ absorption exhibits less ^15^N-isotopic shift (9.1 cm^–1^) than the Pr–N stretching mode of NPrO (22.3 cm^–1^), thus implying that the 826.3 cm^–1^ absorption can instead be assigned as an antisymmetric NPrO stretching mode. Calculations also suggested that the symmetric stretching is too weak to be observed.

Theoretical calculations were performed to elucidate the structure, oxidation states and bonding of the observed species. Geometry optimizations were performed at both the B3LYP and CCSD(T) levels of theory on the neutral NPrO molecule with spin multiplicity of 1, 3 and 5, and the results are listed in Table 2. Calculations at both levels of theory show that the singlet and triplet electronic states are linear while the quintet state is bent. Both levels of theory show that the quintet state is much higher in energy than the other two spin states. The triplet state is predicted to be 7.5 kcal mol^–1^ lower in energy than the singlet state at the B3LYP level, but is 10.0 kcal mol^–1^ higher than the latter at the more accurate CCSD(T) level. Both the quintet and triplet states can thus be ruled out as they are clearly excited states. The calculated Pr–N and Pr–O stretching vibrational frequencies are compared to the experimental values in Table 1. While the calculated harmonic frequencies are in general higher than the experimental anharmonic frequencies,^31^ the noble-gas matrix effect of neon,^32^ which is not considered here, is another factor that contributes to the difference between the matrix experimental and computed values. Table 1 shows that the vibrational frequencies calculated at the B3LYP level are slightly higher than those calculated at the CCSD(T) level. The calculated values for the singlet state match the experimental values, whereas the calculated frequencies for the triplet state are too low to fit the experimental values. Therefore, the experimentally observed NPrO molecule in solid neon can be confidently assigned to have a singlet electronic ground state.

**Table 2:** Calculated relative energies (kcal mol^–1^), geometries (bond lengths in Å, bond angles in degrees), vibrational frequencies (unscaled, cm^–1^) and intensities (km mol^–1^) of the neutral NPrO molecule at the B3LYP, CCSD(T) and CASPT2 levels of theory

		B3LYP			CCSD(T)			CASPT2	
		Pr(v)	Pr(iv)	Pr(iii)	Pr(v)	Pr(iv)	Pr(iii)	Pr(v)	Pr(iv)
Δ*E*		0.0	–7.5	54.7	0	10.8	43.4		
State		^1^Σ^–^	^3^Φ	^5^A′	^1^Σ^–^	^3^Φ	^5^A′	^1^Σ^–^	^3^Φ
Electron configuration		f^0^σ^2^π^4^	f_φ_^1^σ^1^π^4^	f^2^σ^1^π^3^	f^0^σ^2^π^4^	f_φ_^1^σ^1^π^4^	f^2^σ^1^π^3^	f^0^σ^2^π^4^	f_φ_^1^σ^1^π^4^
Pr–O		1.765	1.805	1.829	1.775	1.810	1.828	1.794	1.811
Pr–N		1.677	1.767	2.361	1.697	1.779	2.242	1.723	1.769
∠OPrN		180.0	180.0	127.4	180.0	180.0	117.1	180.0	180.0
*ν*	Pr–N stretch	1021.1(378)	826.8(284)	330.5(73)	960	819.3	402.1	896.8(401)	818.4(231)
	Pr–O stretch	822.1(194)	736.2(59)	784.6(300)	788.6	723.1	776.9	735.6(97)	784.1(64)
	NPrO bend	157.0(78)	168.4(74)	63.4(54)	125.7	164.9	119.6		
*S* ^2^		0.0	2.01	6.02					
*T*_1_					0.056	0.046	0.032		
*D*_1_					0.167	0.123	0.091		

Similar to the isovalent uranyl ion (UO_2_^2+^) and the PrO_2_^+^ ion, the neutral NPrO molecule in the singlet ground electronic state also prefers a linear structure in order to optimize the overlap of the Pr 5d and 4f orbitals with those of the N and O atoms. The Pr–N and Pr–O bond lengths are predicted to be 1.677 and 1.765 Å, respectively, at the B3LYP level of theory, and CCSD(T) calculations give slightly higher values (1.697 and 1.775 Å). The Pr–N bond length is about 0.14 Å (B3LYP) or 0.12 Å (CCSD(T)) smaller than the sum of the triple-bond covalent radii of Pr and N proposed by Pyykkö *et al.*^33^ The Pr–O bond distance is also shorter than the sum of the triple-bond covalent radii of Pr and O,^33^ but is about 0.08 Å longer than that of the triple bonded PrO_2_^+^ calculated at the same level of theory.^10^ The predicted bond distances suggest that both Pr–N and Pr–O are multiply bonded. Indeed, the Mayer bond orders of 3.1 for Pr–N and 2.1 for Pr–O calculated at the B3LYP level of theory (Table 3) are consistent with the strong multiple Pr–N and Pr–O bonding interactions observed in the neutral NPrO molecule.

**Table 3:** Mayer bond orders and atomic charges of NPrO and NPrO^–^ calculated at the B3LYP/TZ2P level of theory

	Distance (Å)		Bond order	Charge		
				Atom	Mulliken	Natural
NPrO	Pr–O	1.765	2.1	Pr	1.49	1.49
	Pr–N	1.677	3.1	O	–0.70	–0.76
	N–O	3.442	0.1	N	–0.78	–0.73
NPrO^–^	Pr–O	1.933	1.9	Pr	1.29	1.14
	Pr–N	1.790	3.0	O	–1.08	–1.10
	N–O	3.722	0.1	N	–1.20	–1.05

**Fig. 4:**
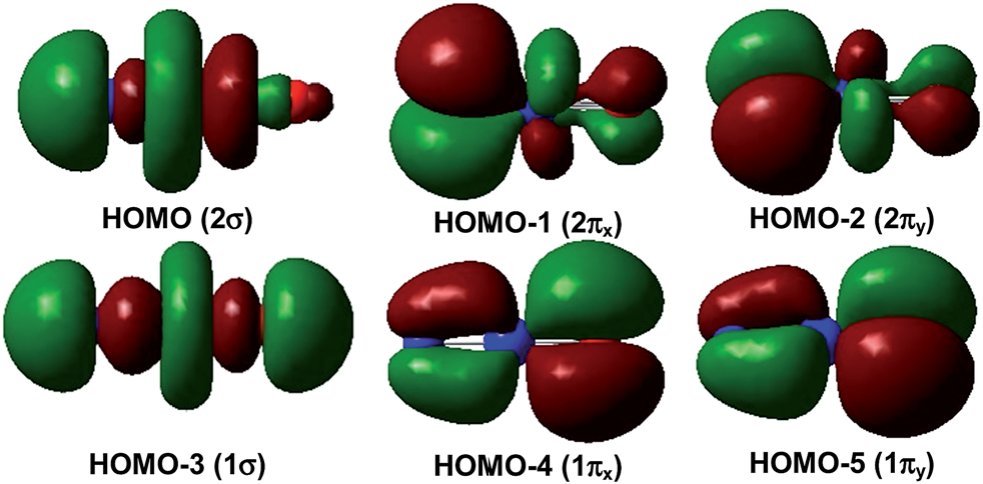
Upper occupied canonical Kohn–Sham valence MO envelopes of the linear singlet NPrO molecule. The 1π orbitals are distributed more on the O atom while the 2σ and 2π orbitals are distributed more on the N atom. The value of the contour envelopes is 0.02 a.u.

The ^1^Σ singlet ground state of NPrO is isoelectronic to PrO_2_^+^ and has a ground electronic configuration of [core](1π)^4^(1σ)^2^(2π)^4^(2σ)^2^(4f5d)^0^. As shown in Fig. 4, the 1σ and 1π MOs are composed of Pr 5d/4f and O (and minor N) 2p atomic orbitals, while the 2σ and 2π MOs are formed by Pr 4f/5d orbitals with N (and minor O) 2p atomic orbitals. All of these six MOs are fully occupied, with both Pr–N and Pr–O bonding characteristics. The wavefunction analyses clearly indicate that the Pr center has a (f^0^d^0^) configuration and that Pr–N is triple bonded and Pr–O is double bonded, which is consistent with the Lewis electron-pair model. Accordingly, the linear singlet NPrO neutral molecule can be classified as a pentavalent praseodymium species with an oxidation state of +V as the PrO_2_^+^ cation. Natural bond orbital (NBO) analyses (see Table S3†) confirm the Pr≡N and Pr=O multiple bonding in NPrO with two sets of localized σ^2^π^4^ bonds. The Pr–N σ bond is composed of 60.9% Pr and 36.8% N character, with a Pr 5d : 4f contribution of 21 : 74 and an N 2s : 2p contribution of 13 : 87. The two degenerate π bonds are each composed of 32.5% Pr and 67.5% N character, with a Pr 5d : 4f contribution of 41 : 58. The Pr–O σ bond is composed of 30.9% Pr and 67.3% O character, with a Pr 5d : 4f contribution of 26 : 71 and an O 2s : 2p contribution of 13 : 87. Each of the two degenerate Pr–O π bonds that represent one covalent and one dative bond are composed of 17.1% Pr and 82.8% O character, with a Pr 5d : 4f contribution of 44 : 55. These bonding analyses clearly show that the Pr–O bonding interaction is much more strongly polarized than the Pr–N bonding interaction, which is consistent with the electronegativity difference and the lower Pr=O bond order. The greater covalency of the Pr–N interaction results in a shorter Pr–N bond (1.677 Å at B3LYP and 1.697 Å at CCSD(T)) than the Pr–O bond (1.765 Å at B3LYP and 1.775 Å at CCSD(T)), albeit the atomic radius of oxygen is slightly smaller than that of nitrogen. As has been discussed before,^10^ the significant covalent bonding interactions of the radially less contracted Pr 5d orbitals with the N and O 2p atomic orbitals in NPrO play an important role in stabilizing the high oxidation state of Pr in these PrO_2_^+^ and NPrO species. In contrast, our preliminary calculations show that the analogous protactinium nitride-oxide NPaO species is a Pa(v) complex with a slightly bent structure because of the significant participation of Pa 5f orbitals in addition to the 6d/7s ones in the chemical bonding.

**Fig. 5:**
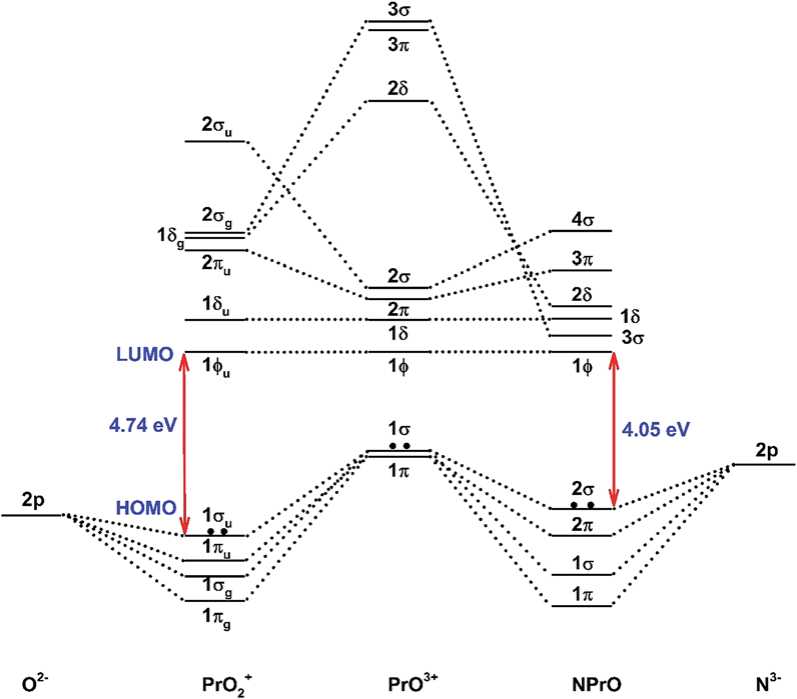
Qualitative energy-level diagram illustrating the correlation of the MOs of PrO_2_^+^ and NPrO in terms of O^2–^ and N^3–^ with PrO^3+^.

Fig. 5 demonstrates a qualitative orbital interaction diagram that shows the correlation of the MOs of PrO_2_^+^ and NPrO, in terms of O^2–^ and N^3–^, with PrO^3+^. In this diagram, only the 2p atomic orbitals of the N and O atoms are shown, with the 2s ones omitted for clarity. The bonding interactions in these two isoelectronic species are in general quite similar. The interactions of the three 2p atomic orbitals of O^2–^ or N^3–^ with the highest fully occupied 1π and 1σ MOs of PrO^3+^ lead to the six highest fully occupied MOs of PrO_2_^+^ and NPrO (Fig. 4 in ref. 10 for PrO_2_^+^ and Fig. 4 for NPrO), all of which possess Pr–O and Pr–N bonding character. The Kohn–Sham energy levels of the three 2p atomic orbitals of N^3–^ are very close to those of the 1π and 1σ MOs of PrO^3+^, whereas the energy levels of the three 2p atomic orbitals of O^2–^ are lower than those of the 1π and 1σ MOs of PrO^3+^. The better match between the interacting orbital energy levels together with the greater radial extension of the N^3–^ atomic orbitals over that of O^2–^ results in better orbital overlap and a Pr–N bond in NPrO that is less polarized than the Pr–O bond in PrO_2_^+^.

The NPrO^–^ anion is predicted to have a doublet ^2^Φ ground state with a linear structure (Table 4). The Pr–O and Pr–N bond lengths are predicted to be 1.933 Å and 1.790 Å at the B3LYP level of theory and 1.951 and 1.808 Å at the CCSD(T) level of theory, respectively. The Pr–N and Pr–O stretching vibrations are predicted to be 813.9 and 636.9 cm^–1^ at the B3LYP level, and at 767.8 and 613.7 cm^–1^ at the CCSD(T) level. The calculated red-shifts from the corresponding modes of neutral NPrO are 192.3 and 174.8 cm^–1^ at the CCSD(T) level and 207.2 and 185.2 cm^–1^ at the B3LYP level, which are in reasonable agreement with the experimentally observed shifts of 195.3 and 138.3 cm^–1^ (for the major site absorptions of NPrO in solid neon) when considering that no matrix effects are accounted for. The NPrO^–^ anion in the ^2^Φ ground electronic state has an electronic configuration of (core) (1π)^4^(1σ)^2^(2π)^4^(2σ)^2^(1φ)^1^. The unpaired electron is located on the 1φ MO that is a non-bonding Pr-based 4f atomic orbital, which is consistent with Mulliken population analysis which shows that the spin density is located on the Pr center. Therefore, the anion can be regarded as being formed by adding an electron to the 1φ LUMO of the neutral NPrO molecule. The extra electron reduces the natural charge of the Pr center from +1.49 in NPrO to +1.14 in the NPrO^–^ anion, which significantly increases the ionic radii and reduces the electrostatic interaction in the anion. The Pr center in the ^2^Φ state of NPrO^–^ has an (f_φ_^1^d^0^) electronic configuration, and thus corresponds to a Pr(iv) oxidation state. Natural bond orbital analyses indicate that despite the Pr(iv) oxidation state in NPrO^–^, the ^2^Φ ground state NPrO^–^ anion is also a pentavalent species with Pr≡N and Pr=O multiple bonds (Table S3†), which are consistent with the calculated Mayer bond orders of 3.0 and 1.9 for the Pr≡N and Pr=O bonds, respectively (Table 3). Another low-lying linear ^2^Δ electronic state of the NPrO^–^ anion with slightly shorter Pr–N and Pr–O bond distances is predicted to be 7.6 kcal mol^–1^ (B3LYP) or 7.0 kcal mol^–1^ (CCSD(T)) higher in energy than the ^2^Φ ground state. This ^2^Δ state NPrO^–^ anion has a Pr(f_δ_^1^d^0^) configuration and an oxidation state of +IV as well. The most stable quartet state with a linear structure, an (f_δ_^1^f_φ_^1^σ^1^) electronic configuration and a Pr(iii) oxidation state is 15.4 (B3LYP) or 43.3 kcal mol^–1^ (CCSD(T)) higher in energy than the ground state.

**Table 4:** Calculated relative energies (kcal mol^–1^), geometries (bond lengths in Å, bond angles in degrees), and vibrational frequencies (unscaled, cm^–1^) of the NPrO^–^ anion

		B3LYP			CCSD(T)			CASPT2
Δ*E*		0.0	7.6	15.4	0.0	7.0	43.3	
OS		Pr(iv)	Pr(iv)	Pr(iii)	Pr(iv)	Pr(iv)	Pr(iii)	Pr(iv)
State		^2^Φ	^2^Δ	^4^H	^2^Φ	^2^Δ	^4^H	^2^Φ
Electron configuration		f_φ_^1^	f_δ_^1^	f_δ_^1^f_φ_^1^σ^1^	f_φ_^1^	f_δ_^1^	f_δ_^1^f_φ_^1^σ^1^	f_φ_^1^
Pr–O		1.933	1.922	1.834	1.951	1.940	1.855	1.950
Pr–N		1.790	1.780	1.794	1.808	1.799	1.826	1.796
∠OPrN		180.0	180.00	180.0	180.0	180.0	180.0	180.0
*S* ^2^		0.82	0.94	3.76				
*ν*	Pr–N stretch	813.9	821.8	740.6	767.8	770.2	728.1	813.9
	Pr–O stretch	636.9	610.8	675.6	613.7	609.4	648.7	645.7
	Bend	75.1	85.1	145.6	87.2	96.3	118.4	
*T*_1_					0.035	0.034	0.051	
*D*_1_					0.094	0.086	0.128	

To assess the potential single-reference nature of the electronic structures of the NPrO and NPrO^–^ species, additional single-point multi-configurational SCF calculations using CASSCF were performed on the optimized structures of the linear singlet neutral NPrO molecule and the doublet NPrO^–^ anion at the B3LYP level of theory. The CASSCF calculations, with 12 electrons in 12 orbitals including six bonding orbitals and six antibonding orbitals (Fig. S4†) on the singlet NPrO, indicate that the wavefunction of NPrO does not exhibit significant multi-reference features. While the Pr(v) (f^0^d^0^) configuration from the CASSCF calculations has a dominated weight of 86.7%, the sum of the natural orbital occupation numbers (NOONs) of the six low-lying orbitals amounts only to 0.29 e^–^ in total. Moreover, the *T*_1_-diagnostic value of 0.056 obtained from the single-determinant CCSD(T) calculation also implies that the wavefunction does not exhibit particularly large multi-reference features (Table 2). Similar calculations on the ^2^Φ doublet state NPrO^–^ anion with 13 electrons in 12 orbitals (Fig. S5†) found that the Pr(iv) (f^1^d^0^) configuration has a dominated weight of 88.8% and that the sum of the NOONs of the five low-lying orbitals amounts only to 0.27 e^–^. These results indicate that the single-reference DFT and CCSD(T) calculations are reasonably reliable, and the assignments of the oxidation states of Pr(v) in neutral NPrO and Pr(iv) in the NPrO^–^ anion are appropriate due to the single reference features of their wavefunctions and the calculated state energies.

In addition to the NPrO and NPrO^–^ molecules, our experiments show that the NPrO molecule further reacts with nitric oxide to form NPrO(NO) and NPrO(NO)_2_ complexes spontaneously upon annealing. The NPrO(NO) complex is predicted to have a doublet ground state with a *C*_s_ symmetry (Fig. S6†), which is 8.9 kcal mol^–1^ lower in energy than the separated NPrO and NO reactants calculated at the B3LYP level. Upon NO coordination, the Pr–N bond is elongated from 1.677 Å to 1.708 Å, while the Pr–O bond is slightly shortened from 1.765 Å to 1.761 Å at the B3LYP level of theory, with the NPrO moiety being close to linearity with a bond angle of 177.0°. The N–O, Pr–N and Pr–O stretching modes are computed as 1883.1, 941.4 and 819.2 cm^–1^, respectively, which are in agreement with the experimental values. The NPrO(NO)_2_ complex is predicted to have a singlet ground state (Fig. S6†), which can be regarded as a complex between NPrO and the (NO)_2_ dimer as the two NO subunits are coupled into N_2_O_4_. This kind of ligand–ligand coupling has been observed in some transition metal nitrosyl cation complexes previously.^35^ Upon coordination of the second NO, the Pr–N bond is further elongated to 1.746 Å, which is quite close to the Pr–O bond length of 1.760 Å. The Pr–N and Pr–O stretching modes are strongly coupled, and are better described as antisymmetric and symmetric stretching modes. The Pr center in both NPrO(NO) and NPrO(NO)_2_ retains the +V oxidation state. It is quite interesting to note that upon *λ* > 800 nm light irradiation, the absorptions of the complexes are eliminated with the production of a weakly perturbed PrO_2_ complex absorption at 747.3 cm^–1^. This observation indicates that Pr(v) is reduced to Pr(iv) and that the complexes are converted to PrO_2_ and N_2_ under near infrared light irradiation.

Observation of the NPrO(NO) and NPrO(NO)_2_ complexes indicates that NPrO is a Lewis acid, which points to the possibility of forming complexes with noble gas atoms. DFT/B3LYP calculations with or without dispersion correction (D3) were thus performed on the complexes formed between NPrO and noble-gas (Ng = Ne, Ar) atoms for comparison. The total Ng binding energies are calculated as the energy change for the process: (NPrO)Ng_*n*_ → NPrO + *n*Ng. The calculated binding energies are listed in Table S2† and the energy curves are presented in Fig. S7.† The B3LYP-D3 results indicate that NPrO can be coordinated by six argon atoms in the first coordination sphere to form the NPrO(Ar)_6_ complex, which is predicted to have a *C*_6v_ structure with all the argon atoms equatorially coordinated to the metal center. The total binding energy of the six argon atoms is 12.7 kcal mol^–1^ with dispersion correction. Upon argon atom coordination, the Pr–N and Pr–O stretching modes red-shifted by 11.9 and 7.9 cm^–1^, respectively. This result implies that the NPrO molecule trapped in a solid argon matrix may be regarded as a matrix-isolated NPrO(Ar)_6_ complex, as in the case of the argon-coordinated CUO molecule.27(a),^34^ Similar calculations on neon complexes indicate that NPrO can also bind five or six neon atoms at the equatorial plane. The NPrO(Ne)_5_ complex has a slightly smaller binding energy (∼0.9 kcal mol^–1^), which might be partially responsible for the different matrix sites of NPrO observed. However, consistent with the much lower coordination ability or Lewis basicity of neon, the binding between Ne atoms and NPrO is rather weak, which leads to a total binding energy of only 7.2 kcal mol^–1^ for the NPrO(Ne)_6_ complex, thus suggesting that the coordination effect of neon atoms compared to argon atoms is somewhat more insignificant.

## Conclusion

The reactions of praseodymium atoms with nitric oxide are studied using matrix-isolation infrared absorption spectroscopy. The ground state Pr atoms react with NO to spontaneously form an inserted NPrO molecule upon annealing in solid neon, with the molecule characterized to have a linear structure with both Pr≡N and Pr=O multiple bonds. Thus, the neutral NPrO molecule is another pentavalent species with a Pr(v) oxidation state, which follows the recently reported PrO_2_^+^ complexes with a Pr(v) center.^10^ An inserted NPrO^–^ anion is also formed *via* electron capture of the neutral molecule during the co-condensation process. Although the anion exhibits quite large red-shifted Pr–N and Pr–O stretching frequencies relative to those of the neutral molecule, the anion is characterized to be another pentavalent praseodymium species with Pr≡N and Pr=O multiple bonds. Especially noteworthy is the Pr(iv) oxidation state of this pentavalent NPrO^–^ complex. Evidence is also presented for the formation of the NPrO(NO) and NPrO(NO)_2_ complexes, which convert to PrO_2_ complexes with a Pr(iv) oxidation state under *λ* > 800 nm light irradiation. Theoretical calculations suggest that NPrO is weakly coordinated by noble gas atoms in solid noble gas matrices. The present study together with our previous work on PrO_2_^+^ and PrO_4_ complexes^10^ has demonstrated that lanthanide compounds with a Ln(v) oxidation state are plausible in both oxides and nitrides. Further investigations into lanthanide oxofluorides, nitride-oxides, halides and carbides would be interesting to explore the undeveloped pentavalent lanthanide chemistry.

## Supplementary Material

Supplementary information

## References

[cit1] (a) Adachi G., Imanaka N. and Kang Z. C., Binary Rare Earth Oxides, Kluwer Academic Publishers, 2004.

[cit2] Riedel S., Kaupp M. (2009). Coord. Chem. Rev..

[cit3] Su J., Li W.-L., Lopez G. V., Jian T., Cao G.-J., Li W.-L., Schwarz W. H. E., Wang L.-S., Li J. (2016). J. Phys. Chem. A.

[cit4] (e) Sroor F. M. A. and Edelmann F. T., Lanthanides: Tetravalent Inorganic, Encyclopedia of Inorganic and Bioinorganic Chemistry, Wiley, Hoboken, 2012.

[cit5] (a) Weast R. C., CRC Handbook of Chemistry and Physics, CRC, Boca Raton, 1982.

[cit6] Prandtl W., Rieder G. (1938). Z. Anorg. Allg. Chem..

[cit7] McCullough J. D. (1950). J. Am. Chem. Soc..

[cit8] Willson S. P., Andrews L. (1999). J. Phys. Chem. A.

[cit9] Su J., Hu S. X., Huang W., Zhou M. F., Li J. (2016). Sci. China: Chem..

[cit10] Zhang Q. N., Hu S. X., Qu H., Su J., Wang G. J., Lu J. B., Chen M. H., Zhou M. F., Li J. (2016). Angew. Chem., Int. Ed..

[cit11] Wang G. J., Zhou M. F. (2008). Int. Rev. Phys. Chem..

[cit12] Becke A. D. (1988). Phys. Rev. A.

[cit13] Frisch M. J., Trucks G. W., Schlegel H. B., Scuseria G. E., Robb M. A., Cheeseman J. R., Scalmani G., Barone V., Mennucci B., Petersson G. A., Nakatsuji H., Caricato M., Li X., Hratchian H. P., Izmaylov A. F., Bloino J., Zheng G., Sonnenberg J. L., Hada M., Ehara M., Toyota K., Fukuda R., Hasegawa J., Ishida M., Nakajima T., Honda Y., Kitao O., Nakai H., Vreven T., Montgomery Jr J. A., Peralta J. E., Ogliaro F., Bearpark M., Heyd J. J., Brothers E., Kudin K. N., Staroverov V. N., Keith T., Kobayashi R., Normand J., Raghavachari K., Rendell A., Burant J. C., Iyengar S. S., Tomasi J., Cossi M., Rega N., Millam J. M., Klene M., Knox J. E., Cross J. B., Bakken V., Adamo C., Jaramillo J., Gomperts R., Stratmann R. E., Yazyev O., Austin A. J., Cammi R., Pomelli C., Ochterski J. W., Martin R. L., Morokuma K., Zakrzewski V. G., Voth G. A., Salvador P., Dannenberg J. J., Dapprich S., Daniels A. D., Farkas Ö., Foresman J. B., Ortiz J. V., Cioslowski J. and Fox D. J., Gaussian 09, Revision C.01, Gaussian, Inc., Wallingford CT, 2010.

[cit14] Watts J. D., Gauss J., Bartlett R. J. (1993). J. Chem. Phys..

[cit15] (b) Werner H. J., et al., MOLPRO, 2012, see http://www.molpro.net.

[cit16] Cao X. Y., Dolg M. (2004). J. Mol. Struct.: THEOCHEM.

[cit17] Schmidt M. W., Gordon M. S. (1998). Annu. Rev. Phys. Chem..

[cit18] Aquilante F., Autschbach J., Carlson R. K., Chibotaru L. F., Delcey M. G., De Vico L., Galvan I. F., Ferré N., Frutos L. M., Gagliardi L., Garavelli M., Giussani A., Hoyer C. E., Li Manni G., Lischka H., Ma D., Malmqvist P. Å., Müller T., Nenov A., Olivucci M., Pedersen T. B., Peng D., Plasser F., Pritchard B., Reiher M., Rivalta I., Schapiro I., Segarra-Marti J., Stenrup M., Truhlar D. G., Ungur L., Valentini A., Vancoillie S., Veryazov V., Vysotskiy V. P., Weingart O., Zapata F., Lindh R. (2016). J. Comput. Chem..

[cit19] Reiher M. (2006). Theor. Chem. Acc..

[cit20] Pierloot K., Dumez B., Widmark P.-O., Roos B. O. (1995). Theor. Chim. Acta.

[cit21] Baerends E. J., et al., ADF2013, SCM, Theoretical Chemistry, Vrije Universiteit, Amsterdam, The Netherlands, 2013, http://www.scm.com.

[cit22] van Lenthe E., Baerends E. J., Snijders J. G. (1993). J. Chem. Phys..

[cit23] van Lenthe E., Baerends E. J. (2003). J. Comput. Chem..

[cit24] Mayer I. (1983). Chem. Phys. Lett..

[cit25] Reed A. E., Weinstock R. B., Weinhold F. (1985). J. Chem. Phys..

[cit26] Willson S. P., Andrews L., Neurock M. (2000). J. Phys. Chem. A.

[cit27] Li J., Bursten B. E., Liang B.-Y., Andrews L. (2002). Science.

[cit28] Andrews L., Zhou M. F., Willson S. P., Kushto G. P., Snis A., Panas I. (1998). J. Chem. Phys..

[cit29] Dekock R. L., Weltner W. (1971). J. Phys. Chem..

[cit30] Zhou M. F., Andrews L., Bauschlicher C. W. (2001). Chem. Rev..

[cit31] Sinha P., Boesch S. E., Gu C. M., Wheeler R. A., Wilson A. K. (2004). J. Phys. Chem. A.

[cit32] Jacox M. E. (2002). Chem. Soc. Rev..

[cit33] Pyykkö P., Riedel S., Patzschke M. (2005). Chem.–Eur. J..

[cit34] Liang B., Andrews L., Li J., Bursten B. E. (2002). J. Am. Chem. Soc..

[cit35] Li Y. Z., Wang L. C., Qu H., Wang G. J., Zhou M. F. (2015). J. Phys. Chem. A.

