# Supplementary material for: Pentavalent lanthanide nitride-oxides: NPrO and NPrO– complexes with N[triple bond, length as m-dash]Pr triple bonds
Source: Chem Sci. 2017 Mar 15;8(5):4035–43. doi: 10.1039/c7sc00710h (PMC5434915; doi:10.1039/c7sc00710h)
Supplement: Supplementary file 1 [file SC-008-C7SC00710H-s001.pdf]

Electronic Supplementary Material (ESI) for Chemical Science.
This journal is © The Royal Society of Chemistry 2017

Supporting Information

The Pentavalent Lanthanide Nitride-Oxides: NPrO and $NPrO^-$ Complexes with N≡Pr Triple Bonds

Shuxian Hu,[1,2] Jiwen Jian,[3] Jing Su,[2] Xuan Wu,[3] Jun Li[2*] and Mingfei Zhou[3*]

[1] Beijing Computational Science Research Center, Beijing 100094, China.
[2] Department of Chemistry and Key Laboratory of Organic Optoelectronics & Molecular Engineering of Ministry of Education, Tsinghua University, Beijing 100084, China.
[3] Collaborative Innovation Center of Chemistry for Energy Materials, Department of Chemistry, Shanghai Key Laboratory of Molecular Catalysis and Innovative Materials, Fudan University, Shanghai 200433, China.

Table S1. Product absorption (cm^{-1}) from co-deposition of laser-ablated Pr Atoms with NO in solid neon.

^{14}NO	^{15}NO	$R_{14/15}$	assignment
926.2/918.5 762.2/755.9	903.9/896.6<br>757.0/750.7	1.0247/1.0244<br>1.0069/1.0069	NPrO
730.9<br>623.9	709.2<br>623.8	1.0306<br>1.0002	$NPrO^-$
1862.6<br>886.6<br>751.6	1829.8<br>868.6<br>744.5	1.0179<br>1.0207<br>1.0095	NPrO(NO)
1825.4<br>1720.6<br>826.3	1793.4<br>1690.2<br>817.2	1.0178<br>1.0180<br>1.0111	$NPrO(NO)_2$
747.3	747.3	1.0000	$PrO_2(N_2)$

Table S2. Total binding energies (kcal/mol) of the $NPrO(Ng)_x$ (Ng = Ne, Ar) and $NPrO(NO)_x$ complexes calculated at B3LYP level with and without the dispersion correction.

	B3LYP			B3LYP-D3		
x	Ne	Ar	NO	Ne	Ar	NO
1	-0.65	-0.86	-8.90	-3.30	-4.45	-13.56
2	-0.76	-1.14	-15.80	-4.06	-6.28	-24.41
3	-0.88	-1.45		-4.81	-8.18	
4	-0.93	-1.66		-5.54	-10.23	
5	-0.96	-1.36		-6.27	-12.11	
6	-0.95	-0.54		-7.19	-12.66	
7	-0.65			-0.52	-0.06	

Table S3. The calculated natural localized molecular orbitals (NLMOs) of NPrO and $NPrO^{-}$.

Species	Type	Occ.	NLMO
NPrO	$\sigma_{Pr\text{-}N}$	2.0	60.9%Pr(21%d+74%f) + 36.8%N(13%s+87%p)
	$\pi_{Pr\text{-}N}$	4.0	32.5%Pr(41%d+58%f) + 67.5%N(100%p)
	$\sigma_{Pr\text{-}O}$	2.0	30.9%Pr(26%d+71%f) + 67.3%O(13%s+87%p)
	$\pi_{Pr\text{-}O}$	4.0	17.1%Pr(44%d+55%f) + 82.8%O(100%p)
$NPrO^{-}$	$\sigma_{Pr\text{-}N}$	2.0	59.8%Pr(36%s+22%d+41%f) + 38.6%N(4%s+96%p)
	$\pi_{Pr\text{-}N}$	4.0	24.3%Pr(55%d+43%f) + 75.7%N(100%p)
	$\sigma_{Pr\text{-}O}$	2.0	21.6%Pr(6%s+18%d+74%f) + 76.9%O(13%s+87%p)
	$\pi_{Pr\text{-}O}$	4.0	13.7%Pr(54%d+42%f) + 86.3%O(100%p)

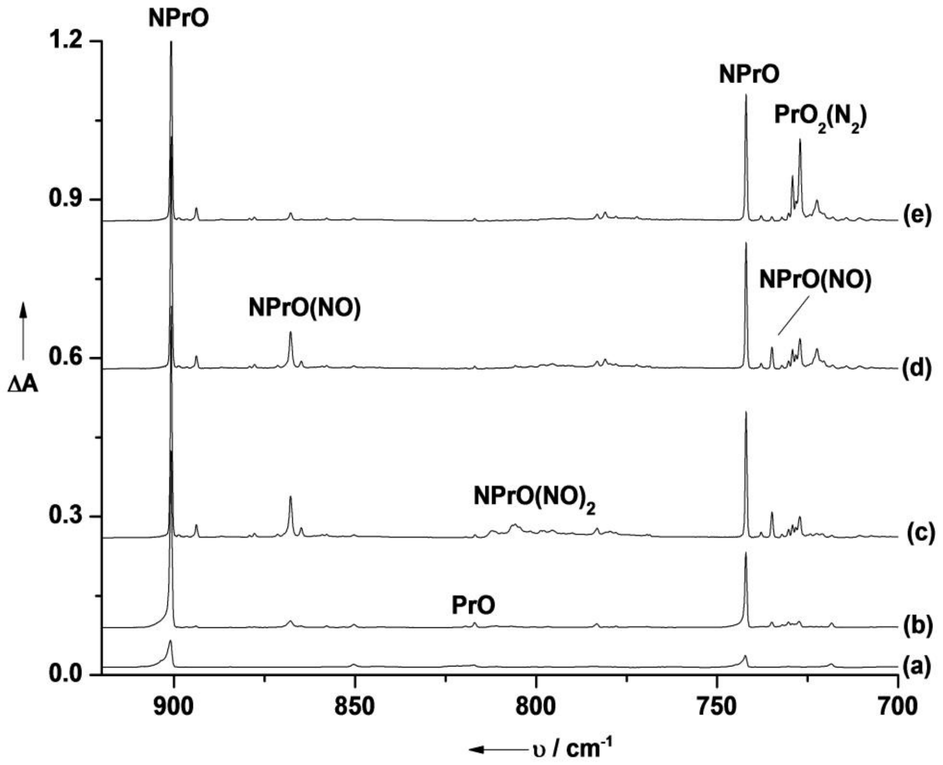

Figure S1. Infrared spectra in the 920-700 cm^{-1} region from co-deposition of praseodymium atoms with 0.1% NO in argon. (a) after 1 h of sample deposition at 4 K, (b) after annealing to 20 K, (c) after annealing to 30 K, (d) after 15 min of l> 800 nm irradiation, and (e) after 15 min of l > 700nm irradiation.

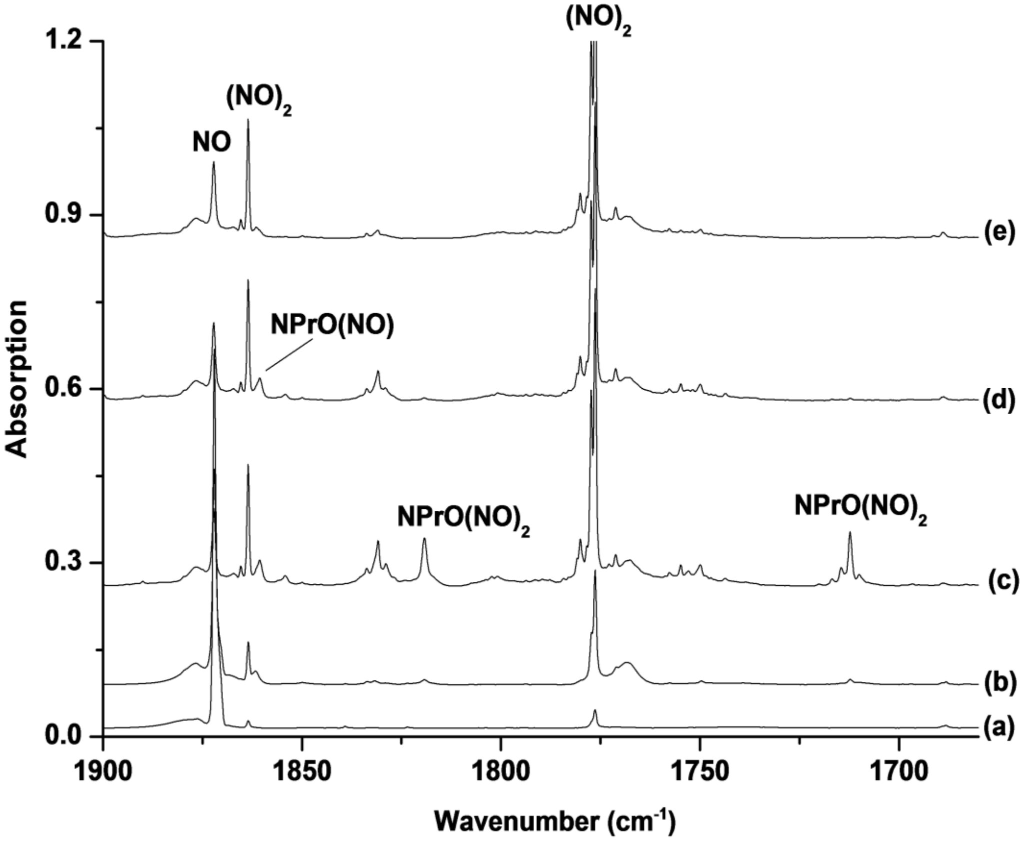

Figure S2. Infrared spectra in the 1900-1680 cm^{-1} region from co-deposition of praseodymium atoms with 0.1% NO in argon. (a) after 1 h of sample deposition at 4 K, (b) after annealing to 20 K, (c) after annealing to 30 K, (d) after 15 min of l > 800 nm irradiation, and (e) after 15 min of l > 700nm irradiation.

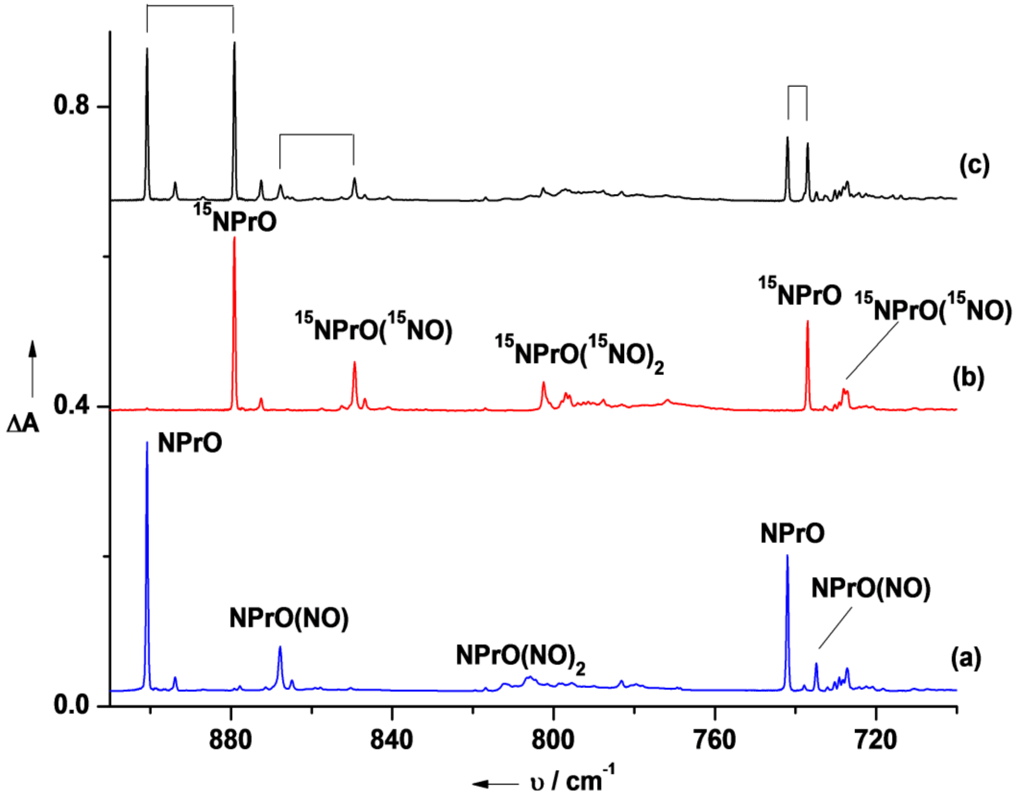

Figure S3. Infrared spectra in the 920-700 cm^{-1} region from co-deposition of praseodymium atoms with isotopic-labeled NO in excess argon. Spectrum was taken after 30 K annealing. (a) 0.1% ^{14}NO, (b) 0.1% ^{15}NO, and (c) 0.05% ^{14}NO + 0.05% ^{15}NO.

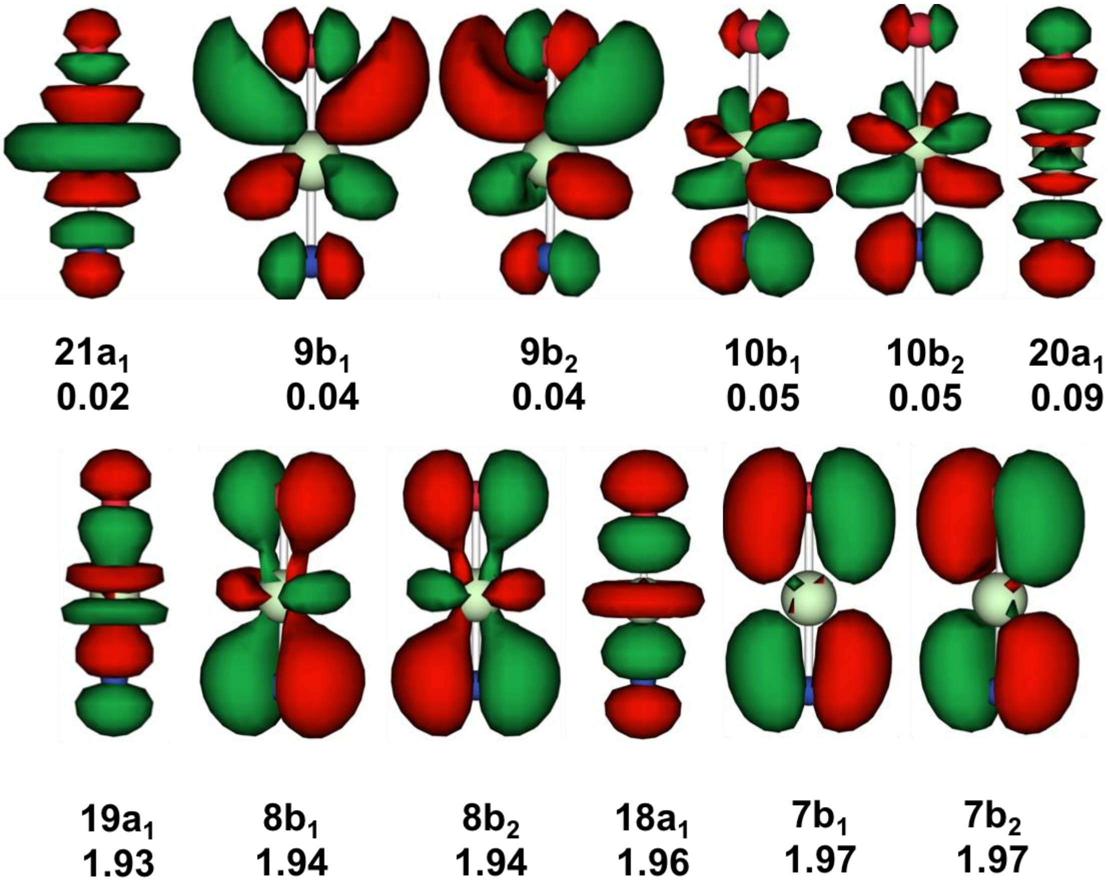

Figure S4. NOONs and natural orbitals of NPrO from CASSCF (12,12)/VDZP calculations.

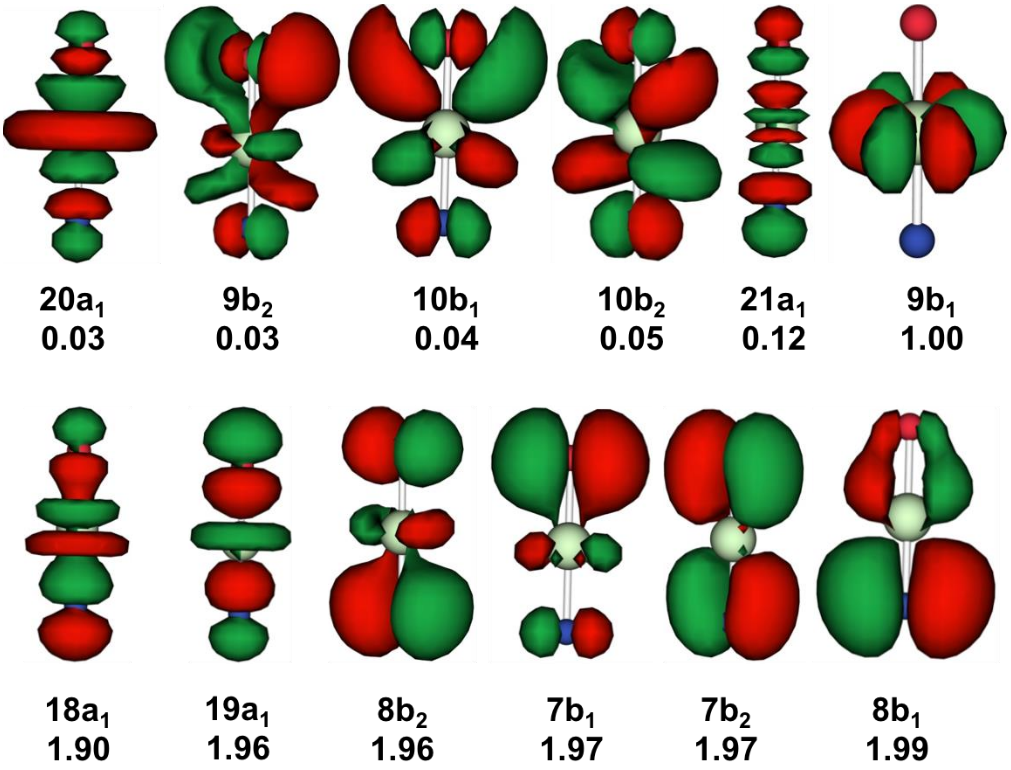

Figure S5. NOONs and natural orbitals of $NPrO^{-}$ from CASSCF (13,12)/VDZP calculations.

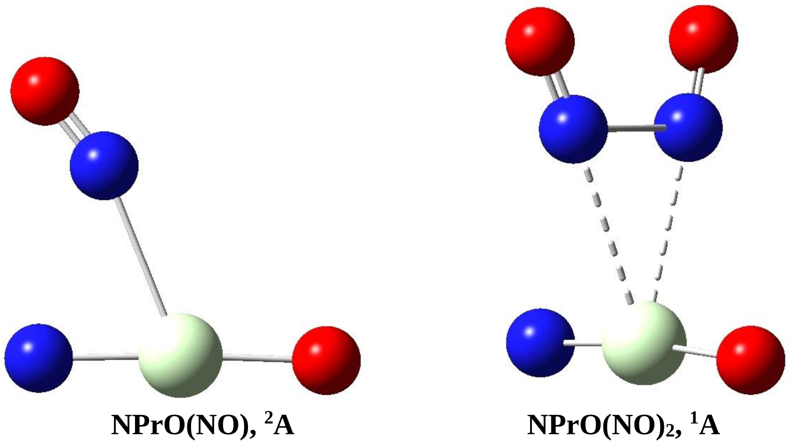

Figure S6. Optimized structures of the NPrO(NO) and $NPrO(NO)_2$ complexes.

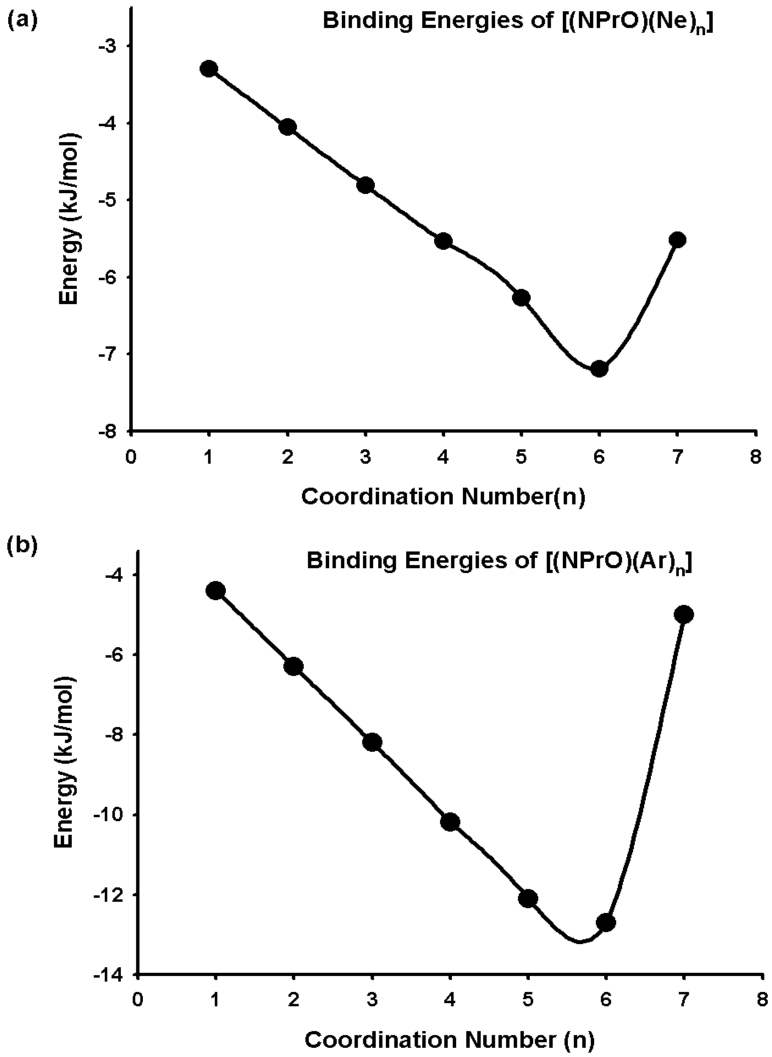

Figure S7. Total binding energies of [NPrO(Ng)$_n$] (Ng = Ne, Ar) calculated at DFT/B3LYP-D3 level.
